# Genome-wide analysis of plant miRNA action clarifies levels of regulatory dynamics across developmental contexts

**DOI:** 10.1101/gr.270918.120

**Published:** 2021-05

**Authors:** Xiaoli Ma, Tom Denyer, Marie Javelle, Antje Feller, Marja C.P. Timmermans

**Affiliations:** 1 Center for Plant Molecular Biology, University of Tübingen, 72076 Tübingen, Germany;; 2 Biogemma, CRC, 63720 Chappes, France

## Abstract

Development of complex organisms requires the delicate and dynamic spatiotemporal regulation of gene expression. Central to this are microRNAs (miRNAs). These mobile small RNAs offer specificity in conveying positional information and versatility in patterning the outcomes of gene expression. However, the parameters that shape miRNA output during development are still to be clarified. Here, we address this question on a genome-wide scale, using the maize shoot apex as a model. We show that patterns and levels of miRNA accumulation are largely determined at the transcriptional level, but are finessed post-transcriptionally in a tissue-dependent manner. The stem cell environments of the shoot apical meristem and vasculature appear particularly liable to this. Tissue-specific effects are also apparent at the level of target repression, with target cleavage products in the vasculature exceeding those of other tissues. Our results argue against a clearance mode of regulation purely at the level of transcript cleavage, leading us to propose that transcript cleavage provides a baseline level of target repression, onto which miRNA-driven translational repression can act to toggle the mode of target regulation between clearance and rheostat. Our data show how the inherent complexities of miRNA pathways allow the accumulation and activity of these small RNAs to be tailored in space and time to bring about the gene expression versatility needed during development.

Multicellular development reflects a delicate succession of cell fate decisions, carefully coordinated in space and time by complex networks of transcription factors (TFs) that convert inputs from positional signals into differential patterns of gene expression. The expression of central TF network components is itself dynamic and precisely regulated to ensure robustness in developmental processes. MicroRNAs (miRNAs) play essential roles in this regulation, and over half of the deeply conserved miRNAs in plants target developmental TFs (Axtell et al. 2007; Cuperus et al. 2011). In addition, conserved miRNA-TF modules have been co-opted repeatedly over the course of evolution for major developmental innovations such as the formation of vasculature, meristems, leaves, roots, flowers, and seed (Plavskin and Timmermans 2012).

miRNA regulation allows for unprecedented versatility in the outcomes of gene expression. In complex with ARGONAUTE (AGO) effector proteins, miRNAs bind in a homology-dependent manner to target transcripts and trigger their site-specific cleavage and/or translational repression (TR) (Yu et al. 2017). On a cellular level, these silencing mechanisms can result in a complete suppression of target accumulation or serve as a rheostat to dampen the noise inherent in gene expression (Voinnet 2009). When mobile positional information coordinates these outcomes on a tissue level, miRNA regulation can promote the formation of spatiotemporal patterns of target gene expression, or when in the rheostat mode, it can refine domains of target activity. Moreover, stemming from their ability to move from cell to cell through plasmodesmata (Vatén et al. 2011), miRNAs can themselves convey the positional information needed to generate developmental patterns (Chitwood et al. 2009; Carlsbecker et al. 2010; Miyashima et al. 2011; Knauer et al. 2013; Skopelitis et al. 2017; Han et al. 2020). As signaling molecules, miRNAs provide a unique degree of specificity and a direct, fully intrinsic mode of action, independent from complex signal transduction pathways, which allows precise and rapid cell fate transitions (Klesen et al. 2020).

The accumulation gradients resulting from the cell-to-cell movement of miRNAs give rise to a variety of patterning effects. For example, xylem cell fates in the root stele are specified according to a miR166 mobility gradient, originating from the endodermis, that establishes an inverse gradient of HD-ZIPIII TF activity (Carlsbecker et al. 2010; Miyashima et al. 2011). However, the opposing miR166 and tasiARF gradients that specify adaxial–abaxial polarity within the leaf generate sharp on-off boundaries of target gene expression via a morphogen-like, threshold-based readout (Skopelitis et al. 2017). The binary shift in gene expression reflects a switch in miRNA activity triggered by the relative levels of small RNA to target that dictates whether miRNAs block target accumulation or show rheostat behavior. The sensitivity with which the miR166 and tasiARF gradients pattern their targets provides a means to flexibly position the adaxial–abaxial boundary in response to environmental or intrinsic cues, while maintaining the required developmental robustness. Finally, mobile small RNA gradients can be tuned to produce stochastic patterns of gene expression (Skopelitis et al. 2017), which are often favored in scenarios in which a bet-hedging of cell fate choices is needed to survive an unpredictable environment. A possible example of this is seen in moss, in which tasiARF generates a stochastic pattern of ARF expression to balance stem cell differentiation in response to environmental cues (Plavskin et al. 2016).

miRNAs thus provide positional information that instructs developmental decisions, and through their versatile regulatory action on targets, lend robustness to developmental programs while allowing for developmental plasticity. The combination of these properties together with their high specificity and rapid mode of action enable the precise regulation of targets across the confined domains commonly found in developmental contexts. Although the contributions of miRNA-directed gene regulation to developmental patterning are quite well established (Voinnet 2009; D'Ario et al. 2017; Klesen et al. 2020), how the accumulation, efficacy, and readout of miRNAs is regulated in space and time to create the necessary developmental precision remains poorly understood. The efficiency of miRNA processing by DICER-LIKE1 (DCL1) is dependent on precursor secondary structure (Bologna et al. 2009). Complementarity is likewise an important variable governing miRNA-target recognition and silencing efficiency (Li et al. 2014a; Liu et al. 2014; Zheng et al. 2017; Agarwal et al. 2018). However, these properties are invariant across development, leaving open the question as to what parameters govern miRNA function in developmental contexts. Here, using the maize shoot apex as a paradigm, we address this question on a genome-wide scale and highlight how the complexity inherent within the miRNA pathway allows regulation of these small RNAs and their action on targets to be tailored to bring about the gene expression versatility needed during development.

## Results

### Subfunctionalization within miRNA families

**Figure 1.**
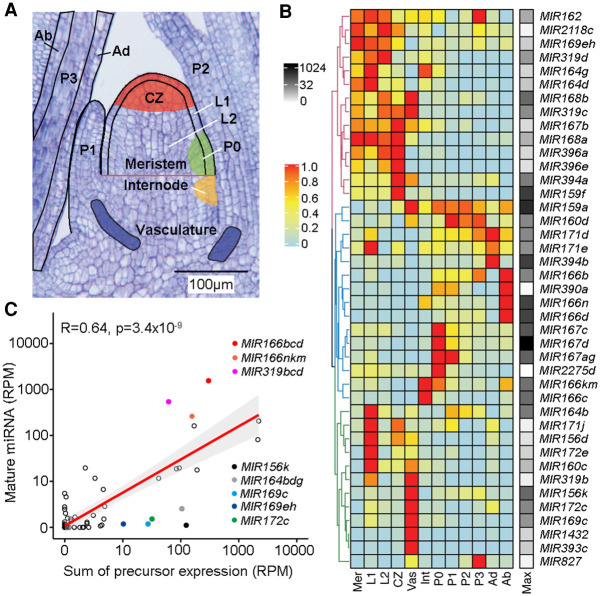
The patterns and levels of miRNA accumulation are principally determined at the transcriptional level. (*A*) Longitudinal section of a B73 maize seedling apex illustrating the 12 domains represented in the expression atlas. Adapted from Knauer et al. (2019). (*B*) Heatmap of 41 miRNA precursors expressed (RPM ≥1) across the maize shoot apex domains. Hierarchical clustering reveals three major clusters. Expression values are normalized per precursor from 0 (lowest value) to 1 (highest value) (Methods). The maximum expression value (RPM) of precursors across all domains is shown to the *right* of the heatmap (black–white scale). (Mer) meristem; (CZ) central zone; (Int) internode; (Vas) vasculature; (Ad) adaxial; (Ab) abaxial; (Max) maximum expression. (*C*) Mature miRNA and precursor expression levels are highly significantly correlated (*r* = 0.64, *P* < 3.4 × 10^−9^). Prominent outliers with higher or lower than expected mature miRNA levels are highlighted.

To understand parameters that shape the spatiotemporal patterns of miRNA activity during development, we took advantage of a recently described high-resolution maize shoot apex expression atlas (Knauer et al. 2019). The SAM is a specialized niche located at the tip of the growing plant shoot that orchestrates the balance between stem cell proliferation and organ initiation essential for post-embryonic shoot growth. The SAM provides a perfect context to study miRNA regulation. Cell fates within the growing niche are continuously defined, and the dynamic expression of key fate determinants is regulated in space and time via miRNA control (Fouracre and Poethig 2016). The expression atlas captures genome-wide expression profiles for the whole meristem, the stem-cell-containing central zone (CZ), the incipient leaf (P0) at the meristem periphery, and the L1 and L2 lineage layers overlaying these meristem regions, as well as for developing leaf primordia P1, P2, and P3, the internode primordium, and vasculature (Fig. 1A; Knauer et al. 2019). Considering the important contributions of small RNAs to adaxial–abaxial leaf polarity (Kuhlemeier and Timmermans 2016), we further expanded the atlas with new expression data for both the adaxial and abaxial sides of P2-P3 leaf primordia (Fig. 1A; Supplemental Fig. S1; Supplemental Data Set S1).

The 28 confirmed maize miRNA families comprise 141 precursor genes (Supplemental Table S1). Although the miRNAs are broadly conserved across land plant evolution, the individual families themselves continue to diverge. The number of precursor genes per family varies relative to other plant species (Baldrich et al. 2018), and single-nucleotide polymorphisms at the level of the mature miRNA are not uncommon (Supplemental Table S1; Supplemental Data Set S1). This is also apparent from their patterns of expression. Together, 41 of the 141 miRNA precursors show expression (≥1 RPM) in at least one of the 12 shoot apex domains (Fig. 1B; Supplemental Data Set S1). For most miRNA families, only a small subset of precursors was detected, pointing to considerable functional diversity among family members (Supplemental Data Set S1).

Those precursors detected in the apex reveal a further division of labor. Hierarchical clustering revealed three major clusters reflecting expression in the meristem, leaf primordia, and vasculature (Fig. 1B). This spatial separation mirrors that previously observed for TFs, which was found to be predictive of tissue identity within the apex (Knauer et al. 2019). Within the main clusters, select defined subclusters point to specialized functions (Fig. 1B; Supplemental Fig. S2A). For instance, several precursors within the miR166 family are expressed primarily on the abaxial side of leaf primordia, consistent with the recorded role of miR166 in specifying abaxial identity (Juarez et al. 2004; Nogueira et al. 2007), and multiple *MIR167* precursors show strong preferential expression in the P0 incipient primordium. However, individual miRNA family members more often show expression in different domains and to substantially different levels (Supplemental Fig. S2). For example, *MIR169c* and *MIR169eh* show complementary expression profiles, marking the vasculature and meristem, respectively. Also, *MIR394b* is strongest expressed on the adaxial side of leaf primordia, whereas *MIR394a,* though expressed here too, is predominantly expressed in the vasculature and CZ (Supplemental Fig. S2B). This augments the presence of substantial functional diversification among miRNA family members. Moreover, the distinct, largely domain-specific expression of miRNA precursors indicates that the spatiotemporal patterns of miRNA activity must result in part from intricate regulation at the transcriptional level. Accordingly, diversification of promoter elements likely caused select precursor genes to fulfill specialized functions in the SAM, vasculature, or leaf primordia. Conversely, it is notable that precursors for a widely diverse set of miRNAs are expressed within each of these domains, pointing to complex combinatorial inputs from multiple miRNAs on cell identity.

### miRNA abundance is largely defined at the transcriptional level

The numerous steps involved in the biogenesis of miRNAs leaves lots of scope for spatiotemporal regulation of miRNA accumulation also at the post-transcriptional level. Complementing RNA-seq with small RNA-seq data provides an opportunity to assess regulation of miRNA accumulation at this level. Accordingly, small RNA-seq was performed on the maize apex, comprising the SAM and up to four-leaf primordia. In total, the 28 maize miRNA families are predicted to produce 76 distinct mature miRNAs (Supplemental Table S1). Forty-one of these miRNAs, spanning 25 families, are detected in the apex (raw read count ≥ 10) (Supplemental Data Set S1). Spearman's correlation analysis revealed that the steady state abundance of most miRNAs is highly significantly correlated (*r* = 0.64, *P* < 3.4 × 10^−9^) to the cumulative level of expression of all its associated precursors across the meristem, internode, P1-P3 leaf primordia, and vasculature; nonoverlapping domains in the apex representing the tissue used in small RNA analysis (Fig. 1C). However, levels of miR166 and miR319 are higher than expected based on expression of their respective precursors, but for the *MIR156k*, *MIR164bdg*, *MIR169c*, *MIR169eh,* and *MIR172c* precursors, mature miRNA accumulation is clearly lower than predicted (Fig. 1C; Supplemental Data Set S1).

**Figure 2.**
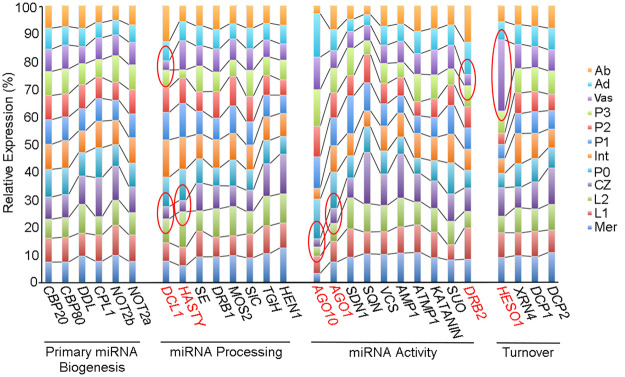
Relative expression of key miRNA pathway genes varies across shoot apex domains. The bar graph illustrates the relative expression of miRNA pathway functions across the 12 apex domains. Although transcript levels for most genes change minimally across the apex, *DCL1*, *HASTY*, *AGO1*, *AGO10*, *HESO1*, and *DRB2* show strong expression variation, particularly in the vasculature and CZ (highlighted red). For functions represented by multiple paralogs in the maize genome, the bar graph is based on cumulative expression of all paralogs (Supplemental Data Set S2).

*MIR156k*, *MIR169c*, and *MIR172c* precursor transcripts are expressed primarily within the vasculature (Fig. 1B; Supplemental Fig. S2B). Most genes required for small RNA biogenesis and turnover show minimal expression variation across the apex (Fig. 2; Supplemental Data Set S2). However, there are a few notable exceptions. Transcript levels for *DCL1* are particularly low in the vasculature and CZ compared to other regions of the apex, whereas relative expression of *HEN1 SUPPRESSOR 1* (*HESO1*), involved in miRNA turnover (Yu et al. 2017), is distinctly higher in the vasculature (Fig. 2). It is conceivable that differences in the activities of these enzymes contribute to spatial regulation of miRNA accumulation, potentially limiting miRNA levels in vascular tissues. Alternatively, or concurrently, the lower than expected levels of miR156k, miR169c, and miR172c are perhaps driven by miRNA mobility. The movement of miRNAs is a carefully regulated process (Rosas-Diaz et al. 2018; Skopelitis et al. 2018), and within the vasculature, regulatory mechanisms are in place to limit the systemic spread of small RNAs. However, small RNAs expressed in the phloem can efficiently move long-distance from the shoot into the root, diminishing their local levels of accumulation (Skopelitis et al. 2018).

Taken together, and analogous to a recent study in animals (Reichholf et al. 2019), miRNA accumulation in maize appears to a large extent determined at the transcriptional level. However, exceptions from this general trend suggest tissue-specific mechanisms are in place to distinctly regulate miRNA accumulation also at a post-transcriptional level.

### Suppression of miRNA accumulation within stem cells of the meristem

**Figure 3.**
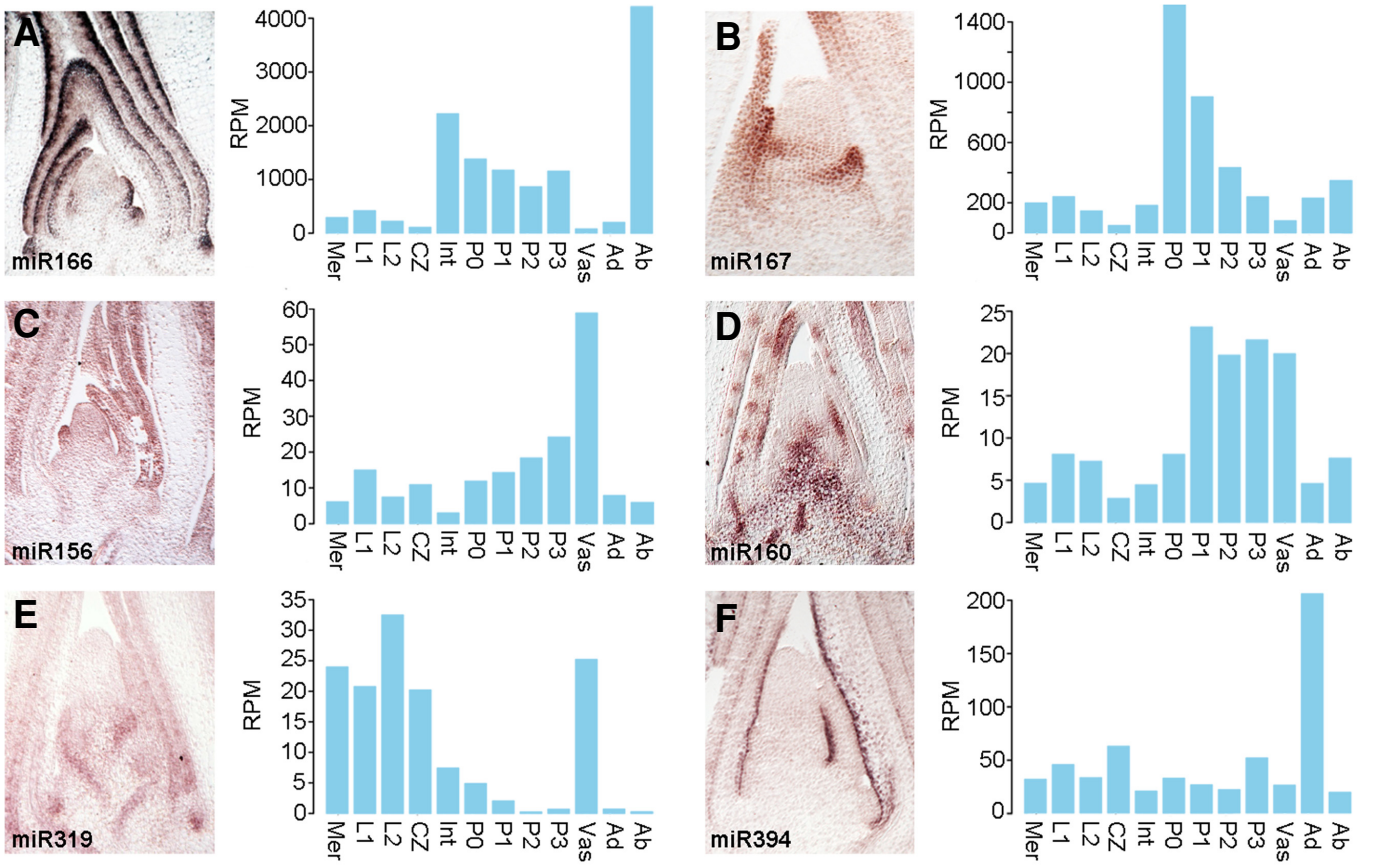
miRNA accumulation within the vasculature and meristem is tuned at the post-transcriptional level: (*A*) miR166; (*B*) miR167; (*C*) miR156; (*D*) miR160; (*E*) miR319; (*F*) miR394. (*A–F*) In situ hybridization patterns of developmentally relevant mature miRNAs in the maize apex (*left*) generally correspond well to the expression profiles of the corresponding precursors (*right*), with the notable exception that miR160 (*D*), miR319 (*E*), and miR394 (*F*) are not detected in the meristem despite prominent precursor expression in those domains.

To further investigate potential tissue-dependent effects on miRNA accumulation on a post-transcriptional level, patterns of mature miRNA localization within the shoot apex were determined by in situ hybridization; focusing on select small RNAs implicated in development (Fig. 3). The accumulation profiles for miR156, miR166, and miR167 across the apex generally correspond well to the cumulative expression patterns of their respective precursors. Specifically, miR166, like its precursors, accumulates in the internode and on the abaxial side of developing leaves, and miR167 is present more broadly in the early primordia (Fig. 3A,B). In addition, the hybridization signal for miR156 is strongest in vascular cells of the stem and leaves, although the pattern of miR156 accumulation extends beyond this tissue and is notably less sharply defined than that of miR160 in the vasculature (Fig. 3C,D). This distinction provides a further indication that miR156 is able to move from its source in the vasculature, consistent with data suggesting non-cell autonomous contributions of miR156 in regulating vegetative phase change in *Arabidopsis* and tuber formation in potato (Bhogale et al. 2014; Fouracre and Poethig 2016). Considering the patterns of directional small RNA mobility within the vasculature (Skopelitis et al. 2018), it follows that miR156 is likely produced in phloem tissues, whereas the more confined accumulation of miR160 predicts it is generated in procambial cells from which small RNAs are unable to move.

In contrast to the aforementioned examples, the accumulation patterns for miR160, miR319, and miR394 in the apex show singular differences to the cumulative expression profiles of their precursors (Fig. 3D–F). miR160, like its precursors, accumulates in the vasculature and leaf primordia, but substantial precursor expression is also seen in the meristem, including the CZ, where mature miR160 is not detected (Fig. 3D). A similar inconsistency is even more pronounced in the profiles of miR319 and miR394. Both miRNAs are not detected in the meristem and CZ, despite substantial precursor expression in these domains (Fig. 3E,F). Indeed, the precursors for miR319 together show near equivalent levels of expression in vascular and meristem tissues, but mature miR319 is only associated with the vasculature (Fig. 3E). Here, expression of miR319 extends also into surrounding cells, suggesting that like miR156, miR319 is mobile and moves from its vascular source into adjacent cells (Fig. 3E).

**Figure 4.**
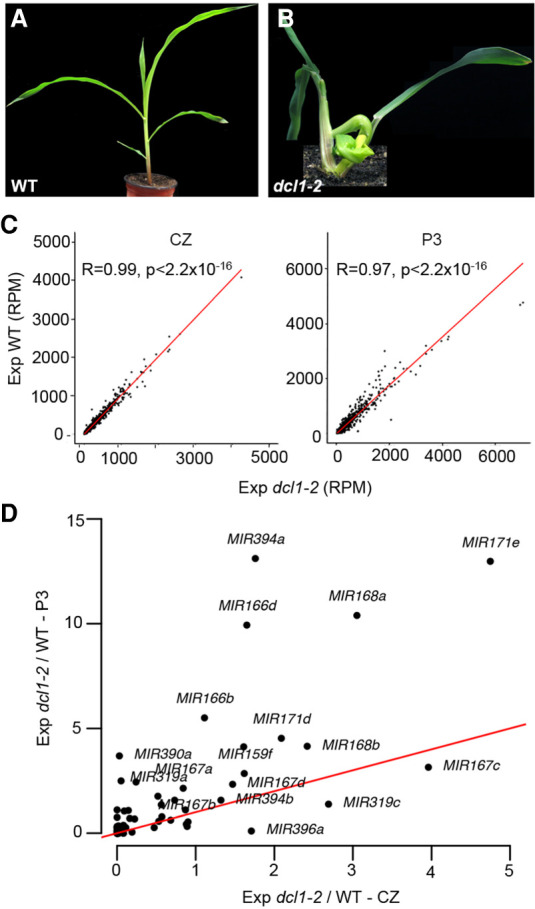
miRNA accumulation within the meristem central zone is suppressed post-transcriptionally by tuning processing efficiency. (*A*,*B*) Compared with the wild type (*A*), 2-wk-old *dcl1-2* (*B*) seedlings show a weak phenotype typified by a compact stature and tightly curled leaves. (*C*) Global gene expression profiles for the CZ (*left*) and P3 primordia (*right*) in *dcl1-2* and wild type are highly significantly correlated (*r* = 0.99 and *r* = 0.97, respectively). (*D*) The effect of *dcl1-2* on precursor levels is far more pronounced in P3 primordia than in the CZ. The red line indicates an equal *dcl1-2*/WT expression ratio in the two tissues. Incidents *above* the red line indicate precursors with higher processing efficacy in P3 primordia.

The meristem and L1 and L2 tissues include cells from the P0 primordium (Fig. 1A). Accordingly, transcripts detected in these meristem samples could conceivably reflect precursor expression in just the incipient leaf. Such an explanation, however, does not hold for the CZ. Instead, miRNA accumulation within the CZ appears to be regulated at the level of biogenesis and/or stability. The observation that *DCL1* transcript levels are comparatively low in the CZ (Fig. 2) is in this regard intriguing. To independently assess regulation of miRNA accumulation in the CZ at the post-transcriptional level, we performed transcriptomic analyses to compare the effect of the *dcl1-2* mutation on precursor expression in the CZ and P3 primordia. The *dcl1-2* hypomorphic allele of *DCL1* was chosen, because, although mature miRNA levels are decreased in *dcl1-2* compared to wild type, its developmental phenotypes are sufficiently mild to minimize potential indirect effects on precursor expression (Fig. 4A,B; Petsch et al. 2015). Indeed, the global gene expression profiles for the CZ and P3 primordia in *dcl1-2* and wild type are highly correlated (*r* = 0.99 and *r* = 0.97, respectively), and the percentages of genes differentially expressed are low (0.43% and 1.20%, respectively; FC ≥ 2, FDR < 0.05) (Fig. 4C). Among the genes up-regulated in the CZ of *dcl1-2* compared to wild type are expressed miRNA precursors, including those of miR319 and miR394. The effect of *dcl1-2* on expression levels varies between precursors, possibly reflecting polymorphisms in secondary structure, which is known to influence processing efficiency (Fig. 4D; Bologna et al. 2009). Moreover, the effect of *dcl1-2* on precursor levels is far more pronounced in P3 primordia (Fig. 4D). This observation, although unable to fully explain the compromised accumulation of miRNAs in the CZ, does point to DCL1-mediated miRNA processing as a major step by which miRNA biogenesis is modulated at the level of individual precursors, as well as at the level of individual tissue types.

Both the levels and spatiotemporal patterns of miRNA accumulation, which are to a large extent determined at the level of precursor transcription, are thus refined at the post-transcriptional level by tuning the processing efficiency, turnover, and mobility of miRNAs. Importantly, processes tune miRNA accumulation in a tissue-dependent manner. It is notable that the starkest differences are seen in the meristem and in the vasculature. Similar to the precisely regulated cell-to-cell movement of small RNAs in the vasculature and stem cell niches (Skopelitis et al. 2018), a multitiered regulation of miRNA abundance seems intuitive considering the many cell fate decisions occurring in these tissues and the precise spatial contexts in which these happen. The meristem and vasculature may therefore be particularly disposed to such dynamic regulation. In line with this notion, cumulative transcript levels for the maize paralogs of *Arabidopsis HASTY, AGO1,* and *AGO10* are likewise substantially lower in the CZ compared to other regions of the apex (Fig. 2).

### miRNA-directed transcript cleavage provides a foundational level of target repression

Besides regulation of the miRNA itself, contributions toward developmental patterning by miRNAs could be further tailored by the manner of target repression. miRNAs repress their targets via transcript cleavage and/or translational repression (TR) (Yu et al. 2017). In addition, miRNA-driven regulation manifests as distinct modes of action, resulting in target clearance or buffering. The former fundamentally leads to mutually exclusive domains of expression, whereas in a rheostat mode, miRNAs are coexpressed with their targets and act to dampen noise in expression and refine domains of activity (Bartel 2009). The morphogen-like patterning properties of mobile small RNAs, such as miR166 and tasiARF, show that miRNA-to-target ratio is a key determinant defining the mode of miRNA regulation. When this ratio exceeds a certain threshold, miRNA activity leads to target clearance, but once this ratio falls below the threshold, regulation switches to a rheostat mode (Skopelitis et al. 2017; Klesen et al. 2020). Whether the miRNA-to-target ratio affects the switch at the level of TR or transcript cleavage and what factors beyond those affecting miRNA-target recognition influence these regulatory mechanisms remain unclear.

To address these questions in the context of the patterning properties of small RNAs, we next studied the relationship between miRNAs and their targets. To identify miRNA targets in the maize apex, a pipeline was produced implementing the TargetFinder tool and degradome (PARE) analysis, which assesses enrichment of 5′ uncapped transcript ends at the predicted cleavage site over chance occurrence (Methods). Altogether, 106 target genes were identified, 104 of which are conserved in *Arabidopsis* (Supplemental Data Set S3). Given the high conservation of targets, an additional 48 target genes were identified by phylogenetic analysis (Supplemental Data Set S3). For 16 miRNA families, both precursor and target transcripts were found present in the apex. For the remaining miRNA families, either one or the other was not detected in any of the tissues sampled. In line with the developmental dynamics of the SAM, the miRNA-target modules identified serve developmental processes, rather than regulating physiological or stress responses (Supplemental Data Set S3).

**Figure 5.**
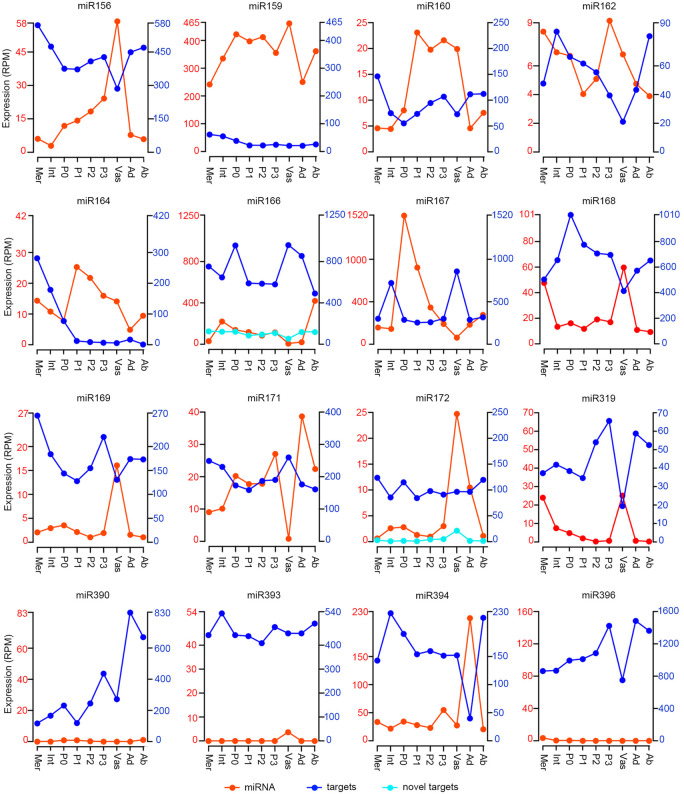
Broad coexpression of miRNA precursors and targets. Plots depicting total precursor and target transcript levels for a given miRNA family across the maize apex domains show considerable coexpression. (*Left* axis in red) Sum expression of all precursors in a miRNA family; (*right* axis in dark blue) sum expression of all targets of the given miRNA family; (light blue lines) expression levels of novel targets (*right*-hand scale). For ease of visualization, only select precursor and target profiles are shown at the same scale; most are shown at a 1:10 miRNA:target scale.

As a first assessment of the regulatory modes and mechanisms used by miRNAs during developmental patterning, we compared the combined transcript levels of targets across the apex domains to those of the miRNA precursors (Fig. 5), which our earlier results indicate serve as a proxy for mature miRNA accumulation (Fig. 1C). From this it is clear that miRNAs are broadly coexpressed with their targets. However, for nearly all miRNAs, both precursor and target transcript levels do fluctuate dynamically across tissues, revealing a degree of expression anti-correlation across domains. This is perhaps best seen in the vasculature where miRNA precursor levels are often highest and target expression is comparatively low (Fig. 5). The miR156, miR162, miR168, miR169, and miR319 families and their targets particularly exemplify this contrast (Fig. 1B; Supplemental Fig. S3). In addition, for miR394, its precursors and targets show complementary expression across the adaxial and abaxial sides of the leaf. Beyond these profiles of grouped targets, the expression profiles of individual targets across the apex reveal also distinct, target-specific variation in the level of coexpression with the miRNA. The SPL, ARF, SCL, and AP2 TF targets of miR156, miR160, miR171, and miR172, respectively, illustrate this (Supplemental Fig. S3).

**Figure 6.**
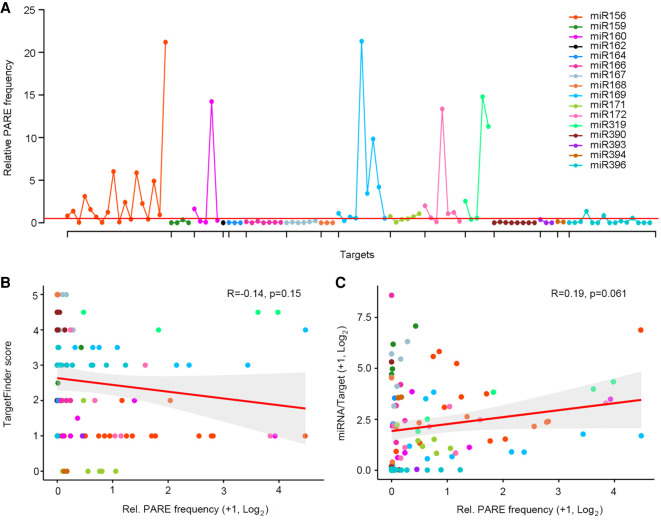
Targets of vasculature-expressed miRNAs show a higher relative PARE frequency. (*A*) Plot of the relative number of PARE signatures for 102 expressed targets shows this number to be low overall. High relative PARE frequencies, reflecting comparatively strong regulation by way of transcript cleavage, are seen for select targets of the vascular miRNAs: miR156, miR160, miR169, miR172, and miR319. The red line indicates a relative PARE value of 0.5. (*B*) The relative number of PARE signatures for natural targets is not correlated to complementarity strength as defined by TargetFinder score. (*C*) The relative PARE frequency of targets is likewise not correlated to the miRNA-to-target expression ratio. The 95% confidence area is marked in gray.

The extensive coexpression of miRNAs with their targets (Fig. 5) seems in line with earlier proposals that few plant miRNAs rely solely on transcript cleavage in repressing their targets (Brodersen et al. 2008; Li et al. 2013a; Yu et al. 2017). However, a potential caveat to this inference is that miRNA-directed cleavage products are generally stable (German et al. 2008; Li et al. 2014a; Schon et al. 2018) and are captured in the overall target transcript levels. As such, to further assess the contribution of miRNA-directed RNA cleavage to developmental patterning, the ratio of PARE signatures over target mRNA levels in the miRNA precursor domain was calculated (Methods). Based on the principles that 3′ cleavage products are stable and not selectively degraded, the relative number of PARE signatures can be used as a comparative metric of cleavage efficacy (Schon et al. 2018; Plotnikova et al. 2019). Analogous to the varying degree of target-miRNA coexpression (Supplemental Fig. S3), this number varies across targets; even targets of the same miRNA (Supplemental Data Set S4). For most targets, the relative PARE frequency was found to be low. This includes targets of miRNAs such as miR164, miR166, and miR171, for which target regulation on a translational level has been shown in *Arabidopsis* (Fig. 6A; Brodersen et al. 2008; Li et al. 2013a). Moreover, of the 102 detected developmental miRNA-target pairs, 11 presented no miRNA-directed degradome products at all, indicating that these targets are not, or are only negligibly, regulated at the level of transcript cleavage (Supplemental Data Set S3).

Taken together, these data provide little evidence for a clearance mode of regulation purely at the level of transcript cleavage, especially considering that some target expression behavior might be explained by promoter dynamics. Instead, the data suggest that much miRNA-driven regulation involves TR and/or serves to buffer the inherent noisiness of gene expression. For miR162 and miR168, which target *DCL1* and *AGO1* transcripts, respectively, a rheostat mode of regulation is to be expected. However, for those targets directing cell fate decisions, the output of miRNA action often includes a clearance mode of regulation (Bartel 2009; Skopelitis et al. 2012, 2017; Klesen et al. 2020). It is therefore intuitive to consider the role of TR among developmental miRNA-target modules.

### Tissue-dependent regulation of target cleavage

Alongside the many examples of low relative PARE frequencies, high PARE-to-transcript ratios, predicting comparatively strong regulation by way of transcript cleavage, are seen for targets of five miRNAs: miR156, miR160, miR169, miR172, and miR319 (Fig. 6A). In each of these miRNA families, a stark divide between targets with very high versus low relative PARE signatures is apparent, consistent with the variation seen in the individual target expression profiles (Supplemental Fig. S3). Regulatory efficiency is affected by a range of factors, including miRNA-target complementarity (Li et al. 2014b). Particularly, base pairing around the AGO1 cleavage site (positions 9–12), is important for miRNA-directed gene regulation, and changes at these positions strongly impact cleavage efficiency (Mallory et al. 2004; Li et al. 2014a). In addition, parameters such as the position and sequence context of the target site have been linked to efficacy of individual miRNA-target interactions (Li et al. 2014a; Liu et al. 2014; Zheng et al. 2017; Agarwal et al. 2018). However, globally, differences in the relative frequencies of PARE signatures in our data are not explained by distinctions in these parameters (Supplemental Data Set S4), and no correlation is evident between PARE-to-transcript ratio and the complementarity “strength” of these natural targets as defined based on TargetFinder score (Fig. 6B).

Likewise, the relative number of PARE signatures is not correlated to miRNA abundance, whether considering targets individually or combined on a per miRNA basis (Supplemental Fig. S4A,B). However, an additional important variable to consider is the contribution of miRNA-to-target ratio. A strongly increased miRNA-to-target level was shown to override the reduced silencing phenotypes of miR159 variants with base substitutions at critical positions (Li et al. 2014a). Moreover, a miRNA-to-target ratio threshold underlies the morphogen-like behavior of mobile small RNAs (Skopelitis et al. 2017). Nevertheless, no correlation between the relative PARE frequency and miRNA-to-target ratio is apparent in our data, either on the level of individual targets (Fig. 6C) or when this ratio is calculated for all targets of a given miRNA combined (Supplemental Fig. S4C), indicating that the morphogenic switch is not programmed at the level of cleavage.

In line with what is seen on a global level, these parameters fail to explain the relative PARE frequency dynamics on a miRNA-intrafamilial, target-to-target level (Fig. 6B,C; Supplemental Data Set S4), with one notable exception. Four miR169 targets with particularly high relative PARE frequencies share a single-nucleotide polymorphism that changes a G:C match into a G:U wobble at the ninth position in the miRNA-target complementary site (Supplemental Data Set S4). This argues that if the earlier observed requirement for base pairing at this position holds true generally (Liu et al. 2014), its effects are regulatory mechanism dependent, which is critical for TR-based repression, but not for cleavage at the adjacent position. Aside from this exception, no direct causative link is evident between the relative frequency of cleavage products for these natural targets and their target site complementarity, target site context, miRNA level, or miRNA-to-target ratio.

It is notable that those miRNAs showing particularly high PARE-to-target ratios are expressed prominently in the vasculature (Figs. 1, 3, and 5; Supplemental Fig. S2). Distinct mechanisms can conceivably explain this observation but considering the reduced coexpression of miRNA precursors and targets in the vasculature (Fig. 5), it seems to predict a unique contribution of miRNA-directed transcript cleavage in this tissue. This point is further reinforced by the low expression of *DSRNA-BINDING PROTEIN 2* (*DRB2*) in the vasculature (Fig. 2). DRB2 is required for TR and also represses fellow DCL1 interaction partner, DRB1, which promotes transcript cleavage (Reis et al. 2015). In addition, transcripts for *HESO1*, which, in association with AGO1, uridylates 5′ miRNA-directed transcript cleavage products to trigger their degradation and thus facilitates miRNA-target-AGO1 complex turnover (Ren et al. 2014), are more abundant in the vasculature (Fig. 2). However, it must be noted that the vasculature is a complex tissue, comprising a mix of cell types that conceivably distinctly affect miRNA-directed target repression. This might account for why the effects on target expression levels in the vasculature appear modest as a whole and vary from target to target (Fig. 5; Supplemental Fig. S3). Our data thus point to tissue type as a prime determinant influencing the level of transcript cleavage and show that the vasculature in particular is distinct from other tissues in this regard.

## Discussion

The number of proteins with recognized roles in miRNA biogenesis and function has grown substantially since the early discoveries of DICER and ARGONAUTE (Yu et al. 2017). Although still largely unexplored, it is easy to envision how the many factors involved could make the miRNA pathway inherently dynamic, allowing miRNA accumulation and activity to be tailored in response to a variable environment or as an aspect of programmed developmental change. Indeed, miRNA regulation allows for unique versatility in the outcomes of gene expression. For instance, as developmental regulators, miRNAs can act as rheostats to lend robustness to developmental programs or serve as positional signals that via a graded, stochastic, or binary readout, generate distinct patterns of cell identity (Miyashima et al. 2011; Plavskin et al. 2016; D'Ario et al. 2017; Skopelitis et al. 2017; Klesen et al. 2020). Here, we investigated parameters that control the accumulation and regulatory outputs of miRNAs in space and time to permit the necessary developmental versatility and precision. Our data highlight the complexity of this regulation and shows that miRNAs, and their action on targets, are regulated at multiple levels in a manner dependent on developmental context.

Reflecting their versatile roles in development, many of the miRNAs that target TFs, F-box proteins, and other developmental regulators have remained conserved throughout land plant evolution (Axtell et al. 2007; Cuperus et al. 2011). In addition, these miRNAs have been co-opted repeatedly over the course of evolution for major innovations, such as the formation of vasculature, meristems, flattened leaves, roots, and seed (Plavskin and Timmermans 2012). Although broadly conserved, individual miRNA families continue to diverge. The evolutionary changes in plant architecture occurred in parallel with repeated gene losses and gains within developmentally important miRNA families (Baldrich et al. 2018). Also, mature miRNAs continue to evolve as evidenced by the frequent occurrence of nucleotide variants within miRNA families. However, as is also apparent from this study, the sets of targets remain largely conserved (Axtell and Bowman 2008). miRNAs are thus co-opted into new developmental contexts along with their targets, and our data show that this is driven by divergence at the promoter level. The patterns of miRNA accumulation we find are largely defined at the transcriptional level, and individual miRNA precursors, even members of a single family, show diverse patterns of expression. Within the maize apex, the select miRNA family members that are expressed show selectivity for either the meristem, leaf primordia, or vasculature. This indicates substantial subfunctionalization within miRNA families, a point that is far less apparent for the targets they regulate.

In addition to the spatiotemporal patterns of precursor expression, levels of miRNA accumulation are determined primarily on a transcriptional level. However, miRNA accumulation patterns are refined post-transcriptionally via tuning at the level of processing, stability, and mobility, in a manner that allows for variability across distinct cell and tissue types. Besides miR166, which is known to move from its source on the abaxial side of leaf primordia to position the adaxial–abaxial boundary (Nogueira et al. 2009), our data predict mobility of miR156 and miR319 in the vasculature. In addition, mechanisms are in place to prevent the accumulation of miRNAs in stem cells of the meristem CZ. These mechanisms act in part via DCL1 and therefore at the level of precursor processing. miRNA processing efficiency is known to be sensitive to parameters affecting precursor secondary structure (Bologna et al. 2009; Moro et al. 2018), predicting some precursor-to-precursor variability. The extent of such variation is apparent in the *dcl1-2* versus wild type transcriptome comparisons. Moreover, these analyses, in accord with reduced *DCL1* expression in the CZ, show that miRNA processing is less efficient in the meristem stem cells as compared to leaf primordia. This situation is distinct from *Arabidopsis,* in which miRNAs not only accumulate, but also repress their targets in the CZ stem cells. In fact, miR394 and miR171 serve as L1-derived positional signals within the *Arabidopsis* CZ that define a domain of stem cell competency and stably anchor this domain to the growing shoot tip (Knauer et al. 2013; Han et al. 2020). Gene expression atlases show that distinct sets of genes characterize stem cell fate in the CZ of maize versus *Arabidopsis* (Knauer et al. 2019; Tian et al. 2019), and our data build on this, demonstrating that miRNA regulation is also distinct in the two meristems.

Beyond regulation of the miRNA itself, our study reveals developmental variation in miRNA-driven target repression. Based on high miRNA-target sequence complementarity, transcript cleavage has historically been considered the principal silencing mechanism used by miRNAs in plants. Our global comparison of miRNA and target expression across domains of the apex together with an overall low relative frequency of cleavage products reveals little evidence for a clearance mode of action driven exclusively by transcript cleavage. This finding is in line with proposals that most plant miRNAs repress their targets using both transcript cleavage and TR (Brodersen et al. 2008; Li et al. 2013a; Yu et al. 2017) and indeed lead us to posit that TR is widespread in miRNA-dependent patterning.

Relative PARE read counts suggest large variation in the contribution of target cleavage between miRNAs. Specifically, miR156, miR160, miR169, miR172, and miR319 emerge as having a notably higher cleavage activity, showing further variation between individual target modules. This aids in explaining earlier observations predicting that miRNAs can use different repression mechanisms even on closely related targets, for example, the effects of miR156 on its *SPL* targets (Xu et al. 2016; He et al. 2018). miRNA-target sequence complementarity and parameters affecting target site accessibility are known to impact miRNA efficacy (Li et al. 2014a; Liu et al. 2014; Zheng et al. 2017). Although complex scenarios can be envisioned in which such sequence-based variation in miRNA-target modules contributes to developmental patterning, we see no evidence for this, except for a position 9 wobble polymorphism in select miR169 target transcripts that unexpectedly is linked to an increased ratio of cleavage products. Moreover, the inferred variations in cleavage activity are not dictated by miRNA level or the miRNA-to-target ratio.

Although unaffected by miRNA and miRNA-to-target levels, cleavage activity may be regulated in a tissue-dependent manner. Distinctly higher PARE signature ratios are observed for miRNAs acting in the vasculature compared to other tissues of the shoot apex, and this is typified by strongly reduced expression of *DRB2* and raised expression of *HESO1*, known to be required for miRNA-driven TR and degradation of transcript cleavage products, respectively (Ren et al. 2014; Reis et al. 2015). It is notable that mechanisms tuning miRNA abundance and action are in play particularly in the meristem and vasculature. In *Arabidopsis*, these tissues use a gating mechanism to regulate the cell-to-cell movement of small RNAs (Skopelitis et al. 2018). Although individual cell types within the vasculature are as yet unresolved, the meristem and vasculature may be particularly disposed to such regulation, with both tissues comprising stem cell niches where multiple cell fates are continuously defined in close spatial and temporal proximity.

Considering transcript cleavage and TR as working side-by-side, the question arises of what the relative contributions of these two silencing mechanisms are to developmental patterning, in particular, because miRNA-to-target ratios do dictate the formation of stochastic patterns in development, as well as the morphogen-like behavior of mobile miRNAs (Plavskin et al. 2016; Skopelitis et al. 2017). The underlying threshold-based readout reflects a switch in AGO activity from a clearance mode to a rheostat mode depending on whether the miRNA-to-target ratio exceeds a given threshold. Our data imply that this switch is not programmed at the level of cleavage, leading to the inference that, although miRNA cleavage activity is clearly variable across miRNAs and miRNA-target modules, this variation is less relevant in shaping the readout of miRNA-driven target regulation. Instead, we propose TR as the master tuner that, beyond a base level of transcript cleavage-driven repression, toggles the mode of miRNA-mediated target regulation between clearance and rheostat to bring about the required versatility in patterning outcomes. Given the current advances in quantitative proteomics, a direct evaluation of miRNA-driven TR at a global level and with the sensitivity and resolution needed may soon be possible, enabling a definitive assessment of this model.

The prominence of miRNAs as regulators of developmental gene expression is intuitive given their unprecedented versatility. However, the very complexity of the miRNA pathways connected to this versatility has made the steps that tailor miRNA activities difficult to identify. Our data shed light on this regulation, highlighting the significance of developmental context for regulating miRNA output. Although miRNA accumulation is largely defined on a transcriptional level, levels are finessed post-transcriptionally, providing refined cell and tissue specificity. Further, the contribution of transcript cleavage to target repression also appears governed at the tissue level. The relative contributions of transcript cleavage and TR to miRNA-driven target repression is an important and open question, and here, our data propose that TR is not only widespread but offers a level of regulatory tuning over a baseline of repression supplied by transcript cleavage that is essential for pattern formation. This two-tier mode of regulation would confer a certain level of assurance or adaptability onto cells, given that TR is in principle reversible. Considering the full spectrum of miRNA targets, future studies should resolve whether miRNAs regulating plant responses to the environment are subject to a similar degree of tailoring.

## Methods

### Plant materials

All analyses were performed on 14-d-old B73 seedlings grown under long day conditions (16 h 24°C light to 8 h 20°C dark cycles). The *dcl1-2* allele (Petsch et al. 2015) was introgressed for four generations into B73 before analysis.

### Laser microdissection, RNA-seq library construction, and sequencing

Tissues of interest were isolated from at least six independent apices for each of two biological replicates by laser microdissection, as described previously (Knauer et al. 2019). Adaxial and abaxial tissues were collected from P2 and P3 leaf primordia of 14-d-old B73 seedling apices. For *dcl1-2* to wild type comparisons, the CZ and P3 leaf primordia were dissected from 14-d-old sibling seedling apices. RNA was extracted as previously described (Knauer et al. 2019) and linearly amplified using the ARCTURUS RiboAmp HS Plus Kit (Thermo Fisher Scientific). RNA-seq libraries were constructed using standard Illumina protocols. All libraries were sequenced on Illumina HiSeq platforms. All sequencing metrics can be found in Supplemental Data Set S5.

### Annotation of miRNA and miRNA biology–related genes

Considering that most miRNA genes remain poorly annotated in the filtered gene set (version FGSv5b+), genomic coordinates for miRNA precursors were manually curated based on available transcriptome data for B73 and Mo17 apices (Li et al. 2013b). miRNA hairpin annotations in miRBase (v21) were mapped back to B73 RefGen_v3, and the location of the precursor transcripts defined based on the distribution of RNA-seq reads at the locus. For miRNA genes for which transcriptome data was not available, the gene model was annotated to cover the miRNA hairpin ±100 nt. Curated precursor locations were used to determine transcript accumulation across tissues of the shoot apex atlas, as previously described (Knauer et al. 2019). Maize genes with functions in small RNA biology were identified based on information from published work, or through identification of maize homologs of known *Arabidopsis* genes using standard homology searches (BLASTP 2.26++) and the paralog search tool in BioMart (https://www.gramene.org) (Tello-Ruiz et al. 2018). Expression values for genes with paralogous functions were combined, and relative expression across the 12 SAM domains determined.

### Gene expression analysis

RNA-seq data for the adaxial and abaxial tissue samples were processed and analyzed as described in Knauer et al. (2019). Briefly, trimmed reads were aligned to the B73 RefGen_V3 using GSNAP (Wu and Nacu 2010), and uniquely mapped reads were used for subsequent analyses allowing two or fewer mismatches every 36 bp and fewer than five bases for every 75 bp. miRNA precursor expression values were calculated for all 12 B73 shoot apex samples based on the annotated precursor models. Target gene expression values were calculated similarly or collected from Knauer et al. (2019). Heatmaps were generated using the R package “ComplexHeatmap.” The clustering method was set to the default “hierarchical clustering.” Expression levels were normalized using the formula *y(*i*) = [*x*(*i*) − min(*x*)]/[max(*x*) − min(*x*)]* where **x*(*i*)* represents gene expression in different subdomains; **i** indicates the different tissues types; *max(*x*)* represents the maximum expression value of a given gene across the different domains; *min(*x*)* represents the minimum expression value of a given gene across the different domains; and *y(*i*)* is the normalized value ranging from 0 to 1.

For *dcl1-2* to wild type comparisons, sequence reads were trimmed using Trimmomatic version 0.36 (Bolger et al. 2014) and aligned to the B73 RefGen_v3 reference genome with TopHat version 2.1.1 (Kim et al. 2013). Read counts per gene were computed based on the B73 genome annotation FGSv5b updated with the curated miRNA precursor models from uniquely mapped reads using HTSeq version 0.9 (Anders et al. 2015). Differential gene expression was determined with DESeq2 (Love et al. 2014) on genes with a mean expression value ≥1 RPM in at least one library using default parameters and an absolute log_2_FC ≥ 1 and *Q* < 0.05 cutoff.

### Small RNA data analysis

Total RNA was extracted from 14-d-old B73 shoot apices comprising the SAM and three- or four-leaf primordia using TRIzol reagent (Invitrogen). RNA was treated with DNase I (Promega), and small RNA-seq libraries prepared from 1.2 µg total RNA using standard Illumina protocols. All libraries were sequenced on Illumina Hi-Seq platforms (for sequencing metrics, see Supplemental Data Set S5). Sequence reads were trimmed using cutadapt version 1.13 (Martin 2011). Trimmed reads (18–26 nt) were aligned to the maize B73 RefGen_v3 genome (AGP_v3) using Bowtie version 1.1.2 (Langmead et al. 2009). No mismatches and a maximum of 20 alignments per read were allowed. Reads matching known structural RNAs (rRNAs, tRNAs, sn-RNAs, and sno-RNAs) identified from the Rfam and GenBank noncoding RNA databases (https://www.sanger.ac.uk/tool/rfam/ and https://www.ncbi.nlm.nih.gov/genbank/, respectively), were removed from further analysis. Remaining sequences were annotated using BEDTools (version 2.25.0) (Quinlan and Hall 2010) based on known miRNAs in miRBase (v21).

For correlation analysis between mature miRNA and precursor abundance, sum expression for all precursors associated with a given mature miRNA isoform across meristem, internode, P1, P2, P3, and vasculature (nonoverlapping tissues in the atlas reflecting the tissue used for small RNA analysis) was calculated. Total precursor levels and miRNA abundance were normalized at log-scale, and the Spearman's correlation calculated in R (R Core Team 2018).

### Small RNA target identification and PARE analysis

Potential targets were predicted using TargetFinder, allowing a maximum score of 5 (https://github.com/carringtonlab/TargetFinder). Predicted targets were validated using PARE (parallel analysis of RNA ends) sequencing data generated from 14-d-old B73 seedling apices comprising the SAM and three- or four-leaf primordia (Dotto et al. 2014). PARE captures the 5′ ends of noncapped transcripts, including miRNA-directed cleavage products. Mean PARE tag abundance for large (31 nt, W_L_) and small (5 nt, W_s_) windows around the predicted miRNA cleavage site was calculated from two biological replicates to assess enrichment of signatures at the miRNA cleavage site over chance occurrence, for instance, signatures stemming from RNA degradation (Dotto et al. 2014). Cleavage sites were filtered to retain only those for which W_s_/W_L_ ≥ 0.75 and W_s_ ≥ 4. Potential target genes without PARE signatures were identified using MapMan annotations of the maize filtered gene set (version FGSv5b+) or the paralog search tool in BioMart (https://www.gramene.org) (Tello-Ruiz et al. 2018). Binding site sequences were extracted from TargetFinder. Binding site positions and flanking sequences were annotated based on transcript information for the filtered gene set version FGSv5b+.

The relative frequency of PARE signatures was calculated based on the W_s_ PARE value of a target over its mRNA level in the miRNA precursor domain (Supplemental Table S2). Only targets with a mean expression value ≥1 RPM in at least one tissue were considered. Owing to target sequence conservation, select PARE signatures map to more than one target. Relative PARE signatures were calculated two ways: first, the observed W_s_ signature frequency was assigned to each target independently; second, the signature frequency was distributed to the respective targets according to their relative transcript abundance in the miRNA precursor domain. The two approaches produced analogous results from which the same inferences and conclusions arise. Data using the first approach are presented.

Pearson's correlations between the relative number of PARE signatures and TargetFinder score, miRNA level, or miRNA-to-target ratio were calculated in R (R Core Team 2018). miRNA-to-target ratios were calculated as the sum of miRNA precursor expression over the level of target expression in the miRNA precursor domain (Supplemental Table S2). The combined miRNA-to-target ratio was calculated as the sum expression of miRNA precursors over the sum expression of all targets in the miRNA precursor domains (Supplemental Table S2).

### In situ hybridization

In situ hybridizations were performed on 14-d-old B73 seedling apices, as in Javelle and Timmermans (2012). Probe concentrations and hybridization parameters are detailed in Supplemental Table S3.

## Data access

All raw and processed sequencing data generated in this study have been submitted to the NCBI Gene Expression Omnibus (GEO; https://www.ncbi.nlm.nih.gov/geo/) under accession number GSE147576. Scripts used for miRNA-target analysis are available as Supplemental Code.

## Supplementary Material

Supplemental Material
