# Supplementary material for: Genome-wide analysis of plant miRNA action clarifies levels of regulatory dynamics across developmental contexts
Source: Genome Res. 2021 May;31(5):811–22. doi: 10.1101/gr.270918.120 (PMC8092011; doi:10.1101/gr.270918.120)
Supplement: Supplemental Material [file supp_31_5_811__index.html]

Genome-wide analysis of plant miRNA action clarifies levels of regulatory dynamics across developmental contexts — Supplemental Material 

# Genome-wide analysis of plant miRNA action clarifies levels of regulatory dynamics across developmental contexts

## Supplemental Material

- Supplemental\_Material.pdf
- Supplemental\_Datasets.xlsx
- Supplemental\_Code.zip
