# Supplementary material for: Genome-wide analysis of plant miRNA action clarifies levels of regulatory dynamics across developmental contexts
Source: Genome Res. 2021 May;31(5):811–22. doi: 10.1101/gr.270918.120 (PMC8092011; doi:10.1101/gr.270918.120)
Supplement: Supplemental Material [file supp_gr.270918.120_Supplemental_Material_.pdf]

Supplemental Information for

Genome-wide analysis of plant miRNA action clarifies levels of regulatory dynamics across developmental contexts

Xiaoli Ma[1*], Tom Denyer[1*], Marie Javelle[2], Antje Feller[1], and Marja C. P. Timmermans#[1]

[1] Center for Plant Molecular Biology, University of Tübingen, Auf der Morgenstelle 32, 72076 Tübingen, Germany

[2] Biogemma, CRC, route d'Ennezat, 63720 Chappes, France

* These authors contributed equally to this work

# Corresponding author: Marja C. P. Timmermans

Email: marja.timmermans@zmbp.uni-tuebingen.de

Tel.: +49 7071 29 78099

Table of Contents

Supplemental Figure 1

Supplemental Figure 2

Supplemental Figure 3

Supplemental Figure 4

Supplemental Table 1

Supplemental Table 2

Supplemental Table 3

Supplemental Datasets 1 - 5 (separate file)

Supplemental Code (separate file)

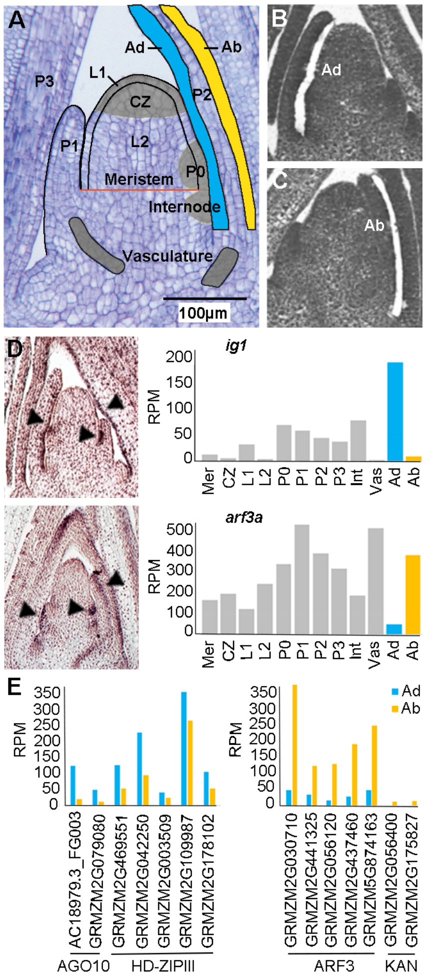

Supplemental Figure 1. Precision of laser microdissection. (*A*) Longitudinal section of a 14-day-old B73 seedling apex highlighting the adaxial (Ad) and adaxial (Ab) sides of the P2 primordium (adapted from Knauer et al. 2019). (*B, C*) Maize apex sections following laser microdissection of the abaxial (*B*) or abaxial (*C*) side of the P2 primordium. (*D*) *in-situ* hybridizations showing localisation of *IG1* and *ARF3a* transcripts on the adaxial and abaxial side of leaf primordia, respectively, match the transcript level profiles in adaxial and abaxial domain RNA-Seq libraries. Black arrowheads highlight select positions of transcript accumulation. (*E*) Expression levels for select marker genes in the adaxial and abaxial domain RNA-seq libraries reflect expectations.

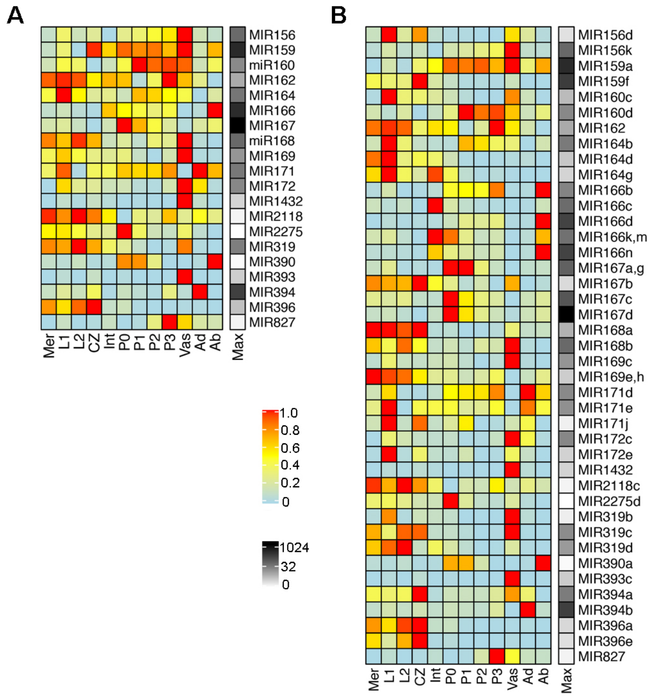

Supplemental Figure 2. Precursor expression is dispersed across SAM domains. *(A, B)* Heatmaps displaying expression profiles across the maize shoot apex for 41 expressed (RPM ≥1 in at least one domain) miRNA precursors combined by family *(A)* and individually *(B)*. The second heatmap is grouped by miRNA family and illustrates that individual members in a family are expressed in different domains and to substantially different levels. Expression values are normalised per precursor or family from 0 (lowest value) to 1 (highest value) (see Methods). The maximum expression value of precursors across all domains is shown to the right of the heatmap (black-white scale).

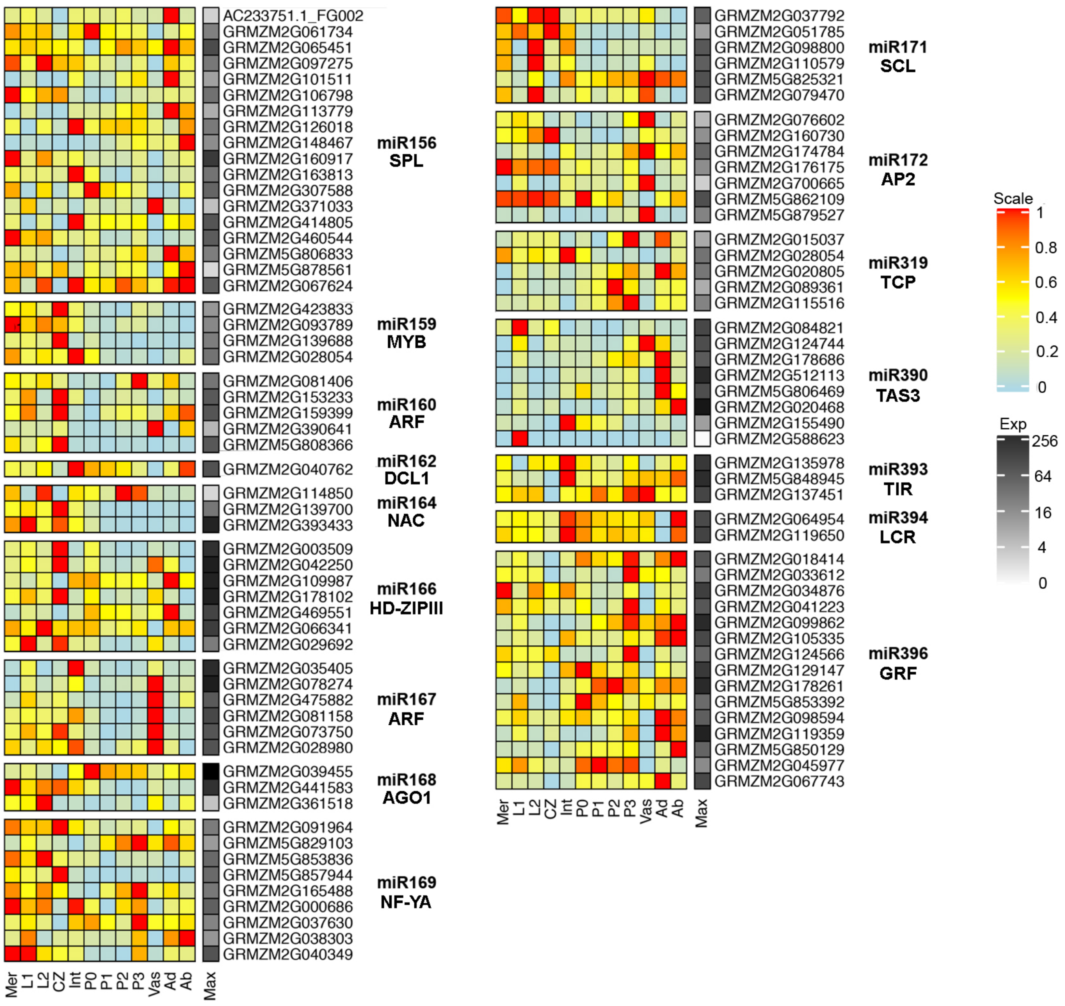

Supplemental Figure 3. Target genes show variable co-expression with their miRNA. Heatmap displaying expression profiles across the maize shoot apex for 102 expressed (RPM ≥1 in at least one domain) targets grouped by miRNA family. Gene expression levels are normalised per target from 0 (lowest value) to 1 (highest value) (see Methods). The maximum expression value of targets across all domains is shown to the right of the heatmap (black-white scale). It is evident that targets show considerable variability in the level of co-expression with their respective miRNA.

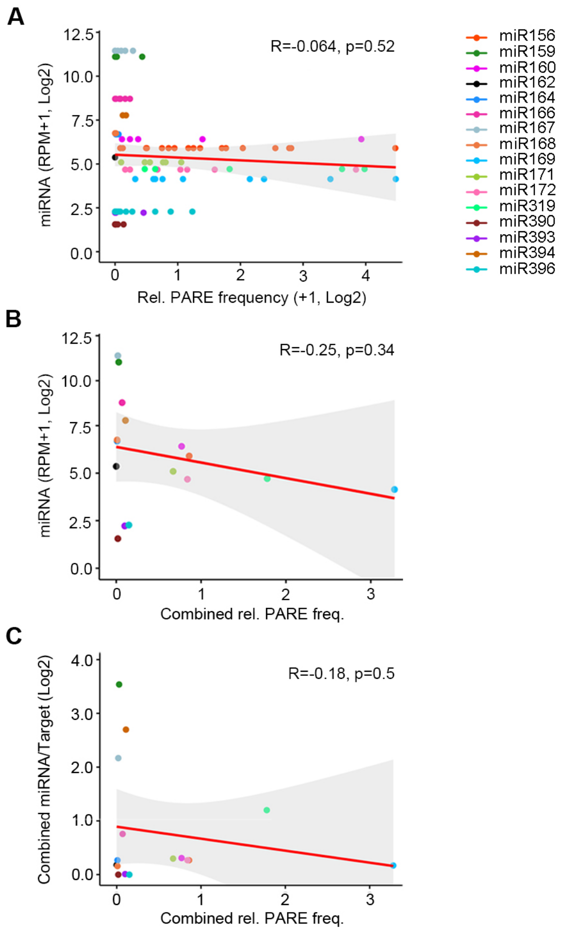

Supplemental Figure 4. Target cleavage efficiency is not dictated by miRNA abundance or miRNA-to-target ratio. (*A, B*) The relative number of PARE signatures for natural targets is not correlated to miRNA abundance, whether considering targets individually (*A*) or combined on a per miRNA basis (*B*). (*C*) Relative PARE frequencies are also not correlated to miRNA-to-target ratio whether on the level of individual targets (**Fig. 6C**), or when this ratio is calculated for all targets of a given miRNA combined, as is shown here. The 95% confidence area is marked in grey.

Supplemental Table 1. Complexity of confirmed maize miRNA families

Family	No. of precursor genes	No. of miRNA hairpins	No. of mature miRNAs
MIR156	12	12	3
MIR159	11	11	5
MIR160	6	6	2
MIR162	1	1	1
MIR164	8	8	4
*MIR166**	11	12	3
*MIR167**	8	9	2
MIR168	2	2	1
*MIR169**	16	17	10
MIR171	11	11	6
MIR172	5	5	2
MIR319	4	4	1
MIR390	2	2	1
MIR393	3	3	1
MIR394	2	2	1
*MIR395**	4	16	4
*MIR396**	7	8	4
MIR397	1	1	1
MIR398	2	2	1
MIR399	10	10	6
MIR408	1	1	1
MIR482	1	1	1
MIR528	2	2	1
MIR529	1	1	1
MIR827	1	1	1
MIR1432	1	1	1
*MIR2118**	5	7	7
*MIR2275**	3	4	4
Total	141	160	76

* miRNA families with polycistronic precursors.

Supplemental Table 2. miRNA precursor domains

miRNA	precursor domain
miR156	vasculature
miR159	meristem, internode, P1, P2, P3, vasculature*
miR160	P1, P2, P3, vasculature
miR162	meristem, internode, P1, P2, P3, vasculature*
miR164	meristem, internode, P1, P2, P3, vasculature*
miR166	abaxial
miR167	P0,P1, P2
miR168	meristem, vasculature
miR169	vasculature
miR171	L1
miR172	vasculature
miR319	vasculature
miR390	meristem, internode, P1, P2, P3, vasculature*
miR393	vasculature
miR394	adaxial
miR396	meristem, internode, P1, P2, P3, vasculature*

*non-overlapping domains of the apex

Supplemental Table 3. ***in situ*** **hybridization probe concentrations and hybridization parameters**

Probe	Sequence	Label	Hybridization temperature	Amount pmol/slide
miR156	GTGCTCACTCTCTTCTGTCA	5'+3'-DIG	50°C	10
miR160	TGGCATACAGGGAGCCAGGCA	5'+3'-DIG	55°C	10
miR166	GGGAATGAAGCCTGGTCCGA	5'-DIG	50°C	10
miR167	TAGATCATGCTGGCAGCTTCA	5'-DIG	55°C	10
miR319	GGGAGCACCCTTCAGTCCAA	5'+3'-DIG	50°C	10
miR394	GGAGGTGGACAGAATGCCAA	5'+3'-DIG	55°C	5
